# Role of vitamin B12 and folic acid in treatment of Alzheimer’s disease: a meta-analysis of randomized control trials

**DOI:** 10.18632/aging.205788

**Published:** 2024-05-02

**Authors:** Chih-Ying Lee, Lung Chan, Chaur-Jong Hu, Chien-Tai Hong, Jia-Hung Chen

**Affiliations:** 1 Department of Neurology, Shuang-Ho Hospital, Taipei Medical University, Taipei, Taiwan; 2 Department of Neurology, School of Medicine, College of Medicine, Taipei Medical University, Taipei, Taiwan

**Keywords:** vitamin B12, folic acid, homocysteine, Alzheimer’s disease

## Abstract

Vitamin B12 and folic acid could reduce blood homocysteine levels, which was thought to slow down the progression of Alzheimer’s disease (AD), but previous studies regarding the effect of vitamin B12 and folic acid in treatment of AD have not reached conclusive results. We searched PubMed and Embase until January 12, 2023. Only randomized control trials involving participants clearly diagnosed with AD and who received vitamin B12 and folic acid were enrolled. Five studies that met the criteria were selected for inclusion in the meta-analysis. Changes in cognitive function were measured based on either the Mini-Mental State Examination (MMSE) or the Alzheimer’s Disease Assessment Scale-Cognitive Subscale (ADAS-Cog). Changes in daily life function and the level of blood homocysteine were also investigated. After a 6-month treatment, administration of vitamin B12 and folic acid improved the MMSE scores more than placebo did (SMD = 0.21, 95% CI = 0.01 to 0.32, *p* = 0.04) but did not significantly affect ADAS-Cog scores (SMD = 0.06, 95% CI = −0.22 to 0.33, *p* = 0.68) or measures of daily life function. Blood homocysteine levels were significantly decreased after vitamin B12 and folic acid treatment. Participants with AD who received 6 months of vitamin B12 and folic acid supplementation had better MMSE scores but had no difference in ADAS-Cog scores. Daily life function did not improve after treatment.

## INTRODUCTION

Alzheimer’s disease (AD) is a progressive neurological disorder characterized by a decline in cognitive function, memory loss, and changes in behavior and personality [1]. It is the most common neurodegenerative disorder worldwide and the leading cause of dementia [2]. From 1990 to 2016, the global prevalence of AD increased 117%, and mortality from AD increased 148% [3]. With these increases come additional economic expenses, AD and AD-related dementia were estimated to cost 2.8 trillion USD in 2019 and are expected to have annual costs of 4.7 trillion and 16.9 trillion USD by 2030 and 2050, respectively [4]. This economic burden is especially large in low- and middle-income countries, which are estimated will bear 65% of all AD costs in 2050 [4]. Therefore, how to slow the progression or reverse the course of AD is imperative and critical.

Currently, there is no cure for AD, and available treatments can only temporarily alleviate symptoms rather than halt or reverse disease progression. Although the cholinesterase inhibitor (ChEI) is the established method of treating AD, it is not always effective in improving cognitive function or slowing disease progression. Many subjects may experience side effects such as nausea, diarrhea, and dizziness, and not all subjects respond well to these medications [5, 6]. Additionally, ChEIs do not address the underlying pathology of AD, such as the accumulation of beta-amyloid plaques and tau tangles in the brain. Hence, there is a need for alternative treatments that can target the underlying disease mechanisms and improve the overall outcomes for subjects with AD. This includes immunotherapies, neuroprotective agents, and lifestyle interventions that can help slow disease progression and improve quality of life for subjects with AD.

Vitamin B12 and folic acid have been discovered to possess neuroprotective properties, as well as being vital for the growth and functioning of the nervous system [7]. Deficiency of both vitamins has been linked to neurodegenerative disorders such as AD, Parkinson’s disease, and multiple sclerosis [8–12]. Therefore, consuming adequate amounts of vitamin B12 and folic acid through a balanced diet or supplements is considered to help protect the nervous system and prevent such conditions. However, there is currently no conclusive evidence that vitamin B12 and folic acid can effectively treat or prevent AD. Some studies have suggested that low levels of vitamin B12 and folic acid may be linked to cognitive decline and an increased risk of developing Alzheimer’s disease, but more research is needed to confirm this association [8]. This meta-analysis analyzed published randomized control trials (RCTs) to explore the effects of vitamin B12 and folic acid on the treatment of AD; these nutrients may offer a supportive management tool for slowing cognitive decline in people with AD.

## RESULTS

### Search strategy and eligibility criteria

**Figure 1:**
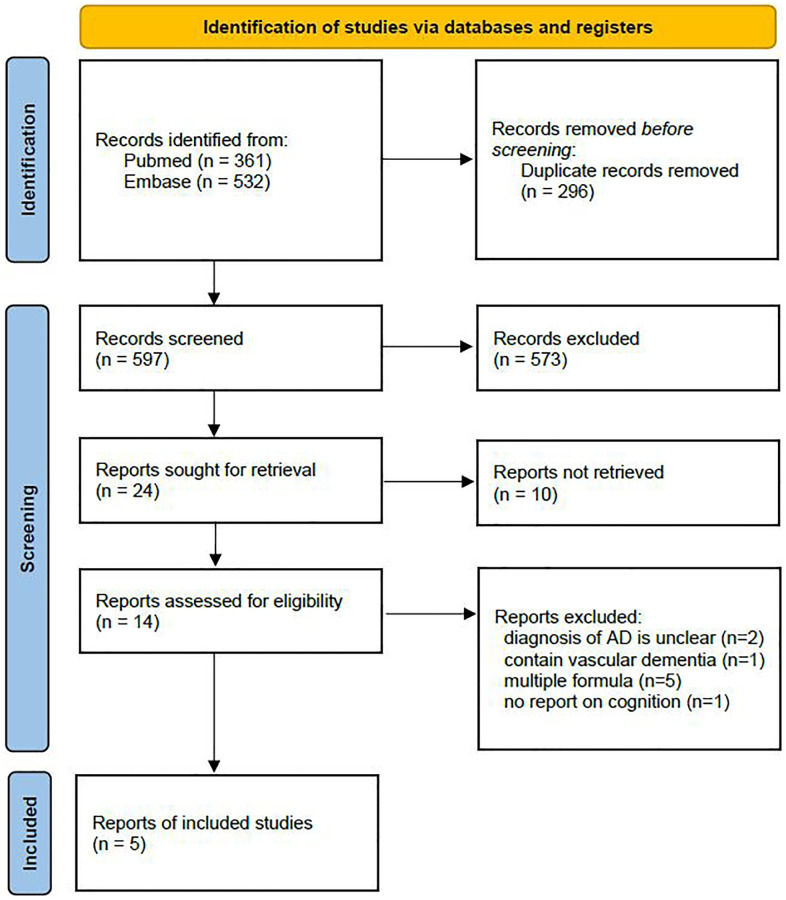
Study selection flow chart.

A total of 893 records were initially identified from PubMed and Embase; 597 remained after duplicates were removed, 24 remained after title and abstract screening, and 14 remained after full-text review. Two of these studies were excluded due to unclear diagnosis of AD, one was excluded because it investigated vascular dementia, five were excluded due to adding other nutrients to their vitamin B12 and folic acid formulas, and another was excluded due to a lack of reporting on the change in cognitive function. In total, five studies were used for the final meta-analysis [13–17]. The study selection flow chart is shown in Figure 1.

Detailed characteristics of the enrolled studies are presented in Table 1. Each of the five studies used randomized, placebo-controlled, and single- or double-blind designs; was published between 2007 and 2021; had a sample size ranging from 41 to 409; and was conducted in Taiwan, the United Kingdom, the United States, or China. Two studies treated participants with only folic acid, and three studies treated participants with vitamin B12 and folic acid. Each study enrolled participants who were clinically diagnosed with AD and had a mean age ranging from 67 to 77 years. Among participants, 429 were allocated to receive vitamin B12 or folic acid, and 351 were allocated to receive placebo; all were under ChEI treatment during the study period. Four studies reported changes in MMSE score, three reported changes in ADAS-Cog score, and four reported changes in daily life functions after 6 months of treatment. All studies measured the change in blood homocysteine levels before and after treatment.

**Table 1:** Characteristics of enrolled studies.

**Author, year**	**Design**	**Inclusion criteria**	**Intervention**	**No. of patients (male, %)**	**Age (mean ± SD)**	**Baseline cognitive function (mean ± SD)**	**Main outcome measure**
Sun, 2007 [13]	RCT-DB	Age >50, mild-to-moderate AD, MMSE 10–26 and CDR 1–2	Mecobalamin 0.5 mg, folic acid 1 mg, and pyridoxine hydrochloride 5 mg for 26 weeks	I: 45 (21, 46.7%) C: 44 (24, 54.5%)	I: 74.9 ± 7.1 C: 74.6 ± 7.5	(I) ADAS-Cog: 24.0 ± 12.3 (I) MMSE: 18.7 ± 4.6 (C) ADAS-Cog: 21.2 ± 10.5 (C) MMSE: 18.6 ± 5.3	Change in ADAS-Cog after 26 weeks
Connelly, 2008 [14]	RCT-DB	Probable AD (fulfilling NINCDS-ADRDA criteria)	Folic acid 1 mg for 6 months	I: 23 (NA) C: 18 (NA)	I: 75.65 ± 5.94 C: 77.60 ± 6.89	(I) MMSE: 23.48 ± 4.10 (C) MMSE: 23.50 ± 2.75	Change in MMSE and IADL after 6 months
Aisen, 2008 [15]	RCT-DB	Age >50, probable AD, MMSE 14–26	Folic acid 5 mg, vitamin B12 1 mg, and vitamin B6 25 mg for 18 months	I: 240 (102, 42.5%) C: 169 (78, 46.1%)	I: 75.7 ± 8.0 C: 77.3 ± 7.9	(I) ADAS-Cog: 22.43 ± 9.0 (I) MMSE: 20.98 ± 3.4 (C) ADAS-Cog: 22.63 ± 8.6 (C) MMSE: 20.91 ± 3.7	Change in ADAS-Cog after 18 months
Chen, 2016 [16]	RCT-SB	Age 40–90, possible or probable AD of mild-to-moderate severity, MMSE 3–26	Folic acid 1.25 mg for 6 months	I: 61 (33, 54.10%) C: 60 (28, 46.67%)	I: 68.10 ± 8.50 C: 67.63 ± 7.92	(I) MMSE: 18.56 ± 6.23 (C) MMSE: 17.63 ± 7.77	Change in MMSE and ADL after 6 months
Chen, 2021 [17]	RCT-SB	Age >45, clinically diagnosed as probable AD, MoCA <22	Folic acid 1.2 mg and vitamin B12 50 μg for 6 months	I: 60 (30, 50.00%) C: 60 (26, 43.33%)	I: 68.58 ± 7.29 C: 68.02 ± 8.34	(I) ADAS-Cog: 24.50 ± 13.79 (*n* = 24) (C) ADAS-Cog: 20.89 ± 9.83 (*n* = 15)	Change in MoCA and ADAS-Cog after 6 months

Abbreviations: RCT: randomized controlled trial; DB: double-blind; SB: single-blind; AD: Alzheimer’s disease; CDR: Clinical Dementia Rating; MoCA: Montreal Cognitive Assessment; I: intervention; C: control; MMSE: Mini-Mental State Examination; ADAS-Cog: Alzheimer’s Disease Assessment Scale-Cognitive Subscale; ADL: Activities of Daily Living; IADL: Instrumental Activities of Daily Living.

### Risk of bias

Each of the five RCTs was determined to have a *low risk of bias* in the randomization process, deviation from the intended interventions, measurement of the outcome, and selection of the reported result. One RCT was classified as having *some concerns* of bias due to missing outcome data, and the others were classified as having *low risk of bias*. The detailed risk of bias assessment is presented in Table 2. There was no publication bias through visual inspection of funnel plots and utilization of Egger’s and Begg’s test, which is provided in the Supplementary Materials section (Supplementary Figures 1–4).

**Table 2:** Risk of bias assessment.

**Author, year**	**Bias arising from the randomization process**	**Bias due to deviations from the intended interventions**	**Bias due to missing outcome data**	**Bias in measurement of the outcome**	**Bias in selection of the reported result**	**Overall risk of bias**
Sun, 2007 [13]	Low	Low	Low	Low	Low	Low
Connelly, 2008 [14]	Low	Low	Low	Low	Low	Low
Aisen, 2008 [15]	Low	Low	Low	Low	Low	Low
Chen, 2016 [16]	Low	Low	Low	Low	Low	Low
Chen, 2021 [17]	Low	Low	Some concerns	Low	Low	Some concerns

### Effect of vitamin B12 and folic acid on cognitive function

**Figure 2:**
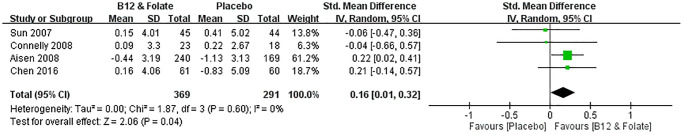
**Effect of vitamin B12 and folic acid on change in MMSE score.** In the pooled treatment group, MMSE score was better than did the pooled control group (SMD = 0.16, 95% CI = 0.01 to 0.32, *p* = 0.04).

**Figure 3:**
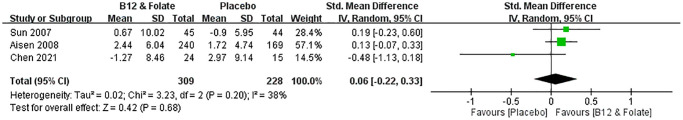
**Effect of vitamin B12 and folic acid on change in ADAS-Cog score.** In both the pooled treatment and control groups, there was non-significant change in ADAS-Cog score after 6-months treatment (SMD = 0.06, 95% CI = −0.22 to 0.33, *p* = 0.68).

The change in cognitive function after vitamin B12 and folic acid treatment after 6 months was measured with the MMSE and ADAS-Cog scales. Among the five included studies, four reported changes in MMSE scores, and three reported changes in ADAS-Cog scores. Improvement in MMSE scores was observed in the treatment arm in three studies, while one study reported a decline in MMSE scores. In the placebo arm, two studies demonstrated improved MMSE scores, while two did not observe this effect. The changes in MMSE scores were found to be lower in the treatment group than in the control group (SMD = 0.2116, 95% CI = 0.01 to 0.32, I2 = 0%, *p* = 0.04, Figure 2). However, the changes in ADAS-Cog scores after 6-months of treatment were non-significant between the treatment and control groups (SMD = 0.06, 95% CI = −0.22 to 0.33, I2 = 38%, *p* = 0.68, Figure 3). Worsening of ADAS-Cog scores was observed in the treatment arm in two studies, as well as in the placebo arm in two studies.

### Effect of vitamin B12 and folic acid on daily life function

**Figure 4:**
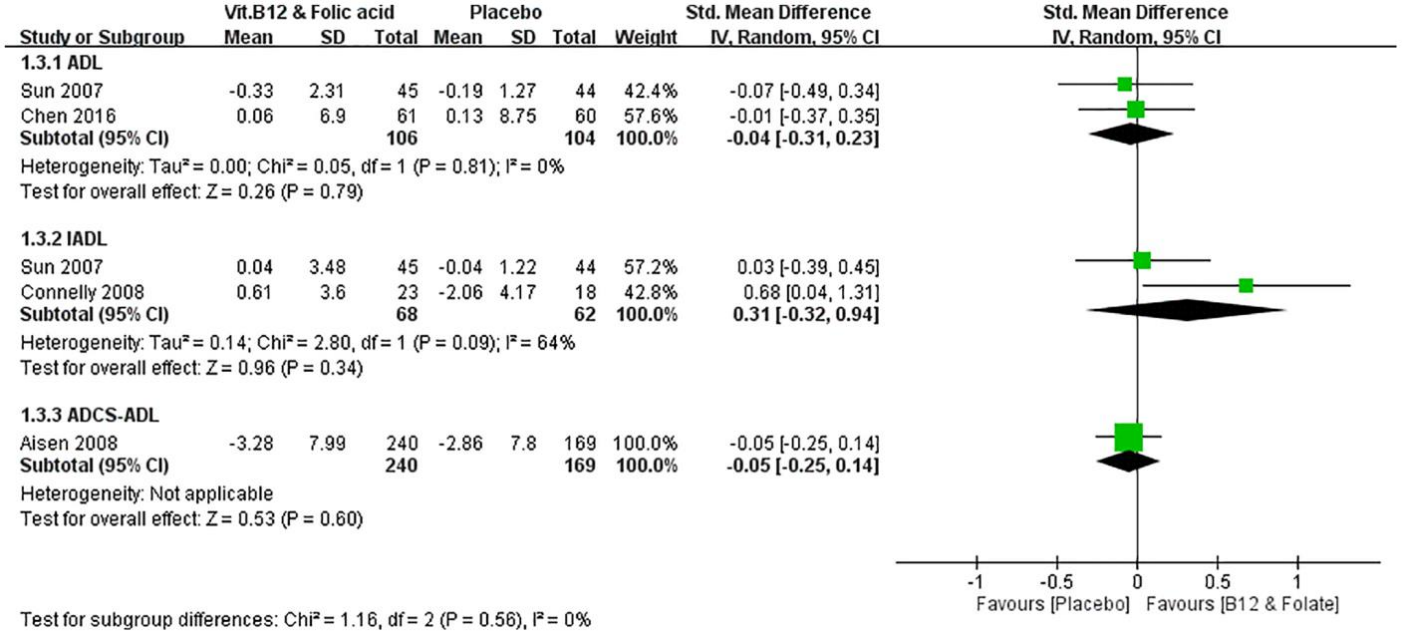
**Effect of vitamin B12 and folic acid on change in daily life functions.** The daily life functions were not significantly different between the pooled treatment and control groups in either ADL (MD = −0.04, 95% CI = −0.31 to 0.23, *p* = 0.79), IADL (MD = 0.31, 95% CI = −0.32 to 0.94, *p* = 0.34), or ADCS-ADL (MD = −0.05, 95% CI = −0.25 to 0.14, *p* = 0.60).

Two studies reported the change in ADL score, two reported the change in IADL score, and one reported the change in Alzheimer’s Disease Co-operative Study-activities of daily living (ADCS-ADL) score. The change in daily life function score in the treatment group was not significantly different from that of the control group after the 6-month treatment, regardless of whether it was measured with the ADL (SMD = −0.04, 95% CI = −0.31 to 0.23, I^2^ = 0%, *p* = 0.79), IADL (SMD = 0.31, 95% CI = −0.32 to 0.94, I^2^ = 64%, *p* = 0.34), or ADCS-ADL (SMD = −0.05, 95% CI = −0.25 to 0.14, *p* = 0.60, Figure 4).

### Change in blood homocysteine level after treatment

**Figure 5:**
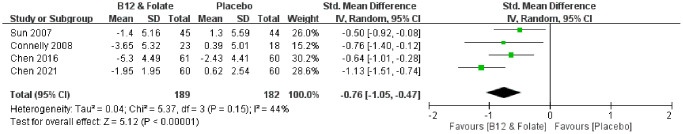
**Effect of vitamin B12 and folic acid on change in blood homocysteine level.** Blood homocysteine level was reduced more significantly in the treatment group than that of the control group (MD = –0.76, 95% CI = –1.05 to –0.47, *p* < 0.00001).

Four studies reported the change in blood homocysteine level before and after the 6-month treatment. The blood homocysteine level in the treatment group was reduced more significantly than that of the control group (SMD = −0.76, 95% CI = −1.05 to −0.47, I^2^ = 44%, *p* < 0.00001, Figure 5).

## DISCUSSION

This meta-analysis included five RCTs that investigated the efficacy of vitamin B12 and folic acid on AD. The results demonstrated that AD subjects receiving vitamin B12 and folic acid exhibited less change in MMSE scores but exhibited no significant difference in ADAS-Cog score. The effect of the vitamin B12 and folic acid intervention on the change in daily life function scores did not differ between the treatment and control groups. The blood homocysteine level significantly decreased after treatment with vitamin B12 and folic acid.

Cognitive impairment is the hallmark and most troublesome symptom of AD. Loss of cholinergic neurons in the nucleus basalis of Meynert, which is found in the early stage of AD, is thought to be responsible for cognitive decline [18, 19]. Vitamin B12 and folic acid play a vital role in many physiological processes, including the development and maintenance of the nervous system. It is particularly important for the development of the cholinergic system, which is essential for memory, attention, and other cognitive functions [20]. There is some evidence to suggest that vitamin B12 may have a modulatory effect on the cholinergic system and could play a role in restoring blood-brain barrier integrity in AD [21]. The blood-brain barrier is a protective layer of cells that separates the brain from circulating blood, and disruptions in this barrier have been linked to the development of AD [22, 23]. Also, folic acid deficiency can lead to a decrease in acetylcholine synthesis and cholinergic neuron degeneration, which can result in cognitive impairment as well [24, 25].

In people with MCI or senile dementia, treatment with vitamin B12 and folic acid has been demonstrated to improve cognitive function. The Folic Acid and Carotid Intima-media Thickness (FACIT) trial enrolled 818 older adults and randomly assigned them to receive folic acid or placebo; taking folic acid supplements for 3 years significantly improved cognitive function [26]. However, Nikita et al. investigated the effect of vitamin B12 and folic acid in 2,919 elderly participants with elevated homocysteine levels. They revealed 2-year supplementation did not enhance cognitive function, although it slightly slowed the rate of cognitive decline [27]. This controversial result was also demonstrated in many studies in which people with AD were treated vitamin B12 and folic acid. In this review, Aisen et al. reported a noteworthy slow decline in MMSE scores after 6 months of treatment, while Sun et al., Connelly et al., and Chen et al. performed three additional RCTs that revealed no significant difference in change of scores [13–16]. Nevertheless, the meta-analysis of all four studies displayed a meaningful less change in MMSE scores for the treatment group. Interestingly, the meta-analysis of three RCTs that reported ADAS-Cog scores showed no significant difference between pretreatment and post-treatment scores [13, 15, 16].

Our findings demonstrated that MMSE scores were influenced by vitamin B12 and folic acid treatment, whereas ADAS-Cog scores were not. One potential explanation for this difference could be attributed to the varying numbers of studies included in the meta-analysis. Specifically, the analysis for MMSE involved four studies, whereas the analysis for ADAS-Cog encompassed only three studies. Although lack of ADAS-Cog scores from the study conducted by Connely et al. may not alter the final result of meta-analysis since its smaller sample size and non-significant difference observed in the change of MMSE scores, it should still be considered as a potential confounding factor. Moreover, subtle disparities were observed in the capacity of MMSE and ADAS-Cog to discern cognitive alterations. While both MMSE and ADAS-Cog assess memory function, MMSE places relatively more emphasis on other cognitive domains, such as orientation, calculation, and visual-spatial skills. The potential influence of vitamin B12 and folic acid on MMSE scores improvement may extend beyond enhancing memory to encompass other cognitive domains. These broader effects could translate into improved MMSE scores.

Despite the MMSE assessing various aspects of cognitive function, the ADAS-Cog offers a more comprehensive evaluation tailored specifically to assess cognitive function in individuals with AD. Previous studies have demonstrated a strong association between the MMSE and the ADAS-Cog, suggesting that they could be utilized for comparable purposes and indicate similar conditions [28, 29]. Although this meta-analysis demonstrated that supplementation of vitamin B12 and folic acid had inconsistent effects in terms of cognitive function assessment, these supplements significantly improved MMSE scores; however, caution is warranted in interpreting these MMSE scores because the better performance was only found in one RCT, which had a larger sample size than others.

The hypothesis that explains why vitamin B12 and folic acid improve cognitive function is that cognitive function is associated with blood homocysteine levels. Vitamin B12 and folic acid are important enzymes cofactors that are essential in remethylation of homocysteine to methionine through methylmalonyl-CoA mutase and methionine synthase [30]. Lack of Vitamin B12 and folic acid lead to elevated blood homocysteine levels, which further cause endothelial dysfunction and vascular injury [31] and result in diseases such as cardiovascular disease, thrombosis, chronic kidney disease, osteoporosis, and neuropsychiatric illness [32–37]. Additionally, altered homocysteine metabolism has been demonstrated to be a consequence of cholinergic disruption, which can be reversed by vitamin B12 supplementation [21]. In all studies of this meta-analysis, treatment with vitamin B12 and folic acid significantly reduced blood homocysteine levels, which was considered to be the mechanism for improving cognitive function, as measured by MMSE performance.

Another hypothesis for why vitamin B12 and folic acid improve cognitive function is related to the pathophysiology of AD. Extracellular deposits of amyloid beta (Aβ) and flame-shaped neurofibrillary tangles are thought to contribute to AD expression [38, 39]. Vitamin B12 and folic acid have been demonstrated to reduce the burden of Aβ deposition in some studies. Lam et al. found vitamin B12 can alleviate mitochondrial fragmentation, bioenergetic defects, and oxidative stress, which protects against Aβ induced proteotoxicity [40]. Li et al. found that folic acid inhibits Aβ peptide accumulation in mice models by enhancing methyltransferase activity [41]. In a study conducted by Chen et al., AD participants receiving folic acid supplementation had a higher Aβ-42 to Aβ-40 ratio [16], which might indicate better cognitive performance because a lower plasma Aβ-42 to Aβ-40 ratio was demonstrated to be associated with greater cognitive decline among older individuals [42].

Although vitamin B12 and folic acid treatment appear to enhance cognitive function in subjects with AD, these nutrients did not significantly improve daily life functions in most studies enrolled in this meta-analysis. In the study conducted by Aisen et al., AD participants exhibited improved MMSE performance after vitamin B12 and folic acid treatment, but ADCS-ADL scores did not improve [15]. Another three studies also demonstrated no difference in the change in daily life functions [13, 16, 17]. However, a study conducted by Connelly et al. reported better IADL scores in the treatment group than in the control group, but cognitive function did not improve in either group [14]. Overall, the results of this meta-analysis did not demonstrate that vitamin B12 and folic acid improve daily functions, although these results might be due to the small number of participants in the studies that were analyzed.

Finally, the studies included in this meta-analysis reported few instances of adverse effects (AEs) after administration of vitamin B12 and folic acid. Sun et al. reported five participants with dizziness, diarrhea, and muscle pain among 45 participants under treatment [13]. Connelly et al. reported no AEs in the treatment group [14]. Participants receiving vitamin B12 and folic acid were reported to have higher levels of depression in a study conducted by Aisen et al. [15]. This effect was opposite to that of previous studies that suggested vitamin B12 and folic acid are beneficial for treating depression [43, 44]. However, the analysis of changes in the depression item of the Neuropsychiatric Inventory scale in their study only showed a trend toward supporting this finding. Consequently, it cannot be conclusively stated that vitamin B12 and folic acid supplementation directly causes depression. Overall, the infrequency of AEs in the included studies suggesting that vitamin B12 and folic supplementation are quite safe.

The strength of this study is its rigorous selection criteria, such as the requirements for a clear diagnosis of AD and randomized control design. The results of this meta-analysis of five RCTs yielded evidence that vitamin B12 and folic acid treatment can improve MMSE scores in subjects with AD, which can be considered for use as an adjuvant therapy. However, this study has several limitations. First, several of the enrolled studies had fewer than 100 participants. The one study that enrolled more cases than others may have skewed the results of the meta-analysis, but this effect was mitigated by the random-effects model used in this study. Second, the reports of clinical outcomes were heterogeneous across studies. Different measurement scales were not able to be entered into the meta-analysis, which limited the number of cases that could be compared and thus limits the power of the results. Third, although vitamin B12 and folic acid are both members of the vitamin B group, they are different substances. In this meta-analysis, we examined the change in blood homocysteine level as mediated by both vitamin B12 and folic acid because we hypothesized that blood homocysteine level is associated with cognitive decline in AD subjects. Finally, vitamin B12 and folic acid dosage variability in the studies may have affected the results. The limited duration of the trials also restricted the outcomes, as a more extended exposure to vitamin B12 and folic acid may be necessary. Yet, despite this variability, all the studies demonstrated decreased blood homocysteine levels, which suggests the related findings are valid.

In conclusion, the administration of vitamin B12 and folic acid in subjects with AD improved MMSE scores, but ADAS-Cog scores and daily life function were not improved. Blood homocysteine level significantly decreased after treatment with vitamin B12 and folic acid. The result should be interpreted with caution due to the use of varying studies for analysis. Further RCTs with larger samples are warranted to provide more evidence on the effect of vitamin B12 and folic acid as AD treatments.

## METHODS

This meta-analysis was performed in accordance with the Preferred Reporting Items for Systematic Reviews and Meta-Analyses guidelines. The review protocol was registered with PROSPERO (CRD42023394167).

### Search strategy and study selection

The literature search and review were conducted by two independent reviewers (C.Y.L. and J.H.C.). PubMed and Embase were searched with no language restrictions until Jan 12, 2023, using keywords related to vitamin B, folic acid, Alzheimer’s, and dementia. The reference sections of prior systematic reviews and meta-analyses were also screened for related studies. The details of the search strategy and results are provided in the Supplementary Materials section (Supplementary Table 1).

Studies were included if they (1) researched a population with a clinical diagnosis of AD, (2) reported on the use of vitamin B12, folic acid, or both as treatment, (3) used a randomized controlled design with at least two comparator arms, and (4) reported outcomes related to changes in cognitive function. All non-RCTs, crossover trials, uncompleted clinical trials, review articles, and studies that did not use original data were excluded. There were no limitations placed on the publication languages. After the duplicate studies were removed, the titles and abstracts of the remaining were screened for eligibility, and the full texts of eligible studies were evaluated.

### Data collection

Baseline characteristics, intervention strategies, and outcome data were independently extracted by two reviewers (C.Y.L. and J.H.C.). Information on study design, study population, and inclusion and exclusion criteria were also retrieved. Disagreements between the two reviewers were resolved through a panel discussion with a third reviewer (C.T.H.) until consensus was reached.

### Outcome measures

The primary outcome was the change in cognitive function after 6 months, which was measured using the Mini-Mental State Examination (MMSE) or the Alzheimer’s Disease Assessment Scale-Cognitive Subscale (ADAS-Cog), of vitamin B12 and folic acid treatment. The secondary outcome was changes in daily life functions, which were measured by the activities of daily living (ADL) scale or the instrumental activities of daily living (IADL) scale. The change in blood homocysteine level after 6 months treatment was also measured.

### Risk of bias assessment

The risk of bias assessment of included studies was performed by two independent reviewers (C.Y.L. and J.H.C.) using version 2 of the Cochrane Risk of Bias Assessment Tool, which categorizes bias risk into *low risk of bias*, *some concerns*, or *high risk of bias*. The publication bias was evaluated through visual inspection of funnel plots and calculated with Egger’s and Begg’s test. Disagreements were resolved through a panel discussion involving all three reviewers (C.Y.L., J.H.C. and C.T.H.).

### Statistical analysis

Analysis was conducted using Review Manager 5.4 (The Cochrane Collaboration, Oxford, UK). The summary effect sizes for outcomes used to compare vitamin B12 and folic acid with placebo were estimated with the DerSimonian and Laird random-effects model. The effect sizes of continuous outcomes are expressed in terms of the standardized mean difference (SMD). The standard deviation (SD) was calculated with the provided confidence interval (CI) limits or interquartile ranges, if the original study SD was not available [45, 46]. Statistical significance was defined by a 95% CI that did not cross zero in the weighted mean difference estimation. Heterogeneity and inconsistency across studies were assessed using the I^2^ statistic.

## Supplementary Materials

Supplementary Figures

Supplementary Table 1
