# Supplementary material for: Role of vitamin B12 and folic acid in treatment of Alzheimer’s disease: a meta-analysis of randomized control trials
Source: Aging (Albany NY). 2024 May 2;16(9):7856–69. doi: 10.18632/aging.205788 (PMC11132008; doi:10.18632/aging.205788)
Supplement: Supplementary Figures [file aging-16-205788-s001.pdf]

SUPPLEMENTARY FIGURES

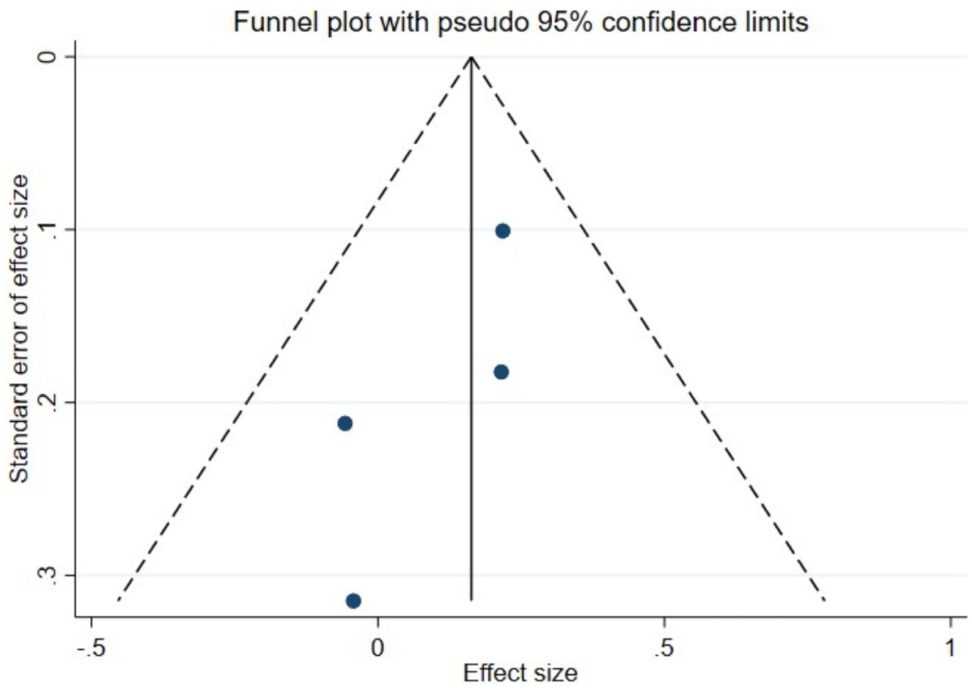

Supplementary Figure 1. Funnel plot of effect of vitamin B12 and folic acid on change in MMSE score. Egger's test (intercept = −1.38, t = −1.84, 2-tailed $p = 0.207$) and Begg's test (z = 1.02, $p = 0.308$) did not reveal any publication bias.

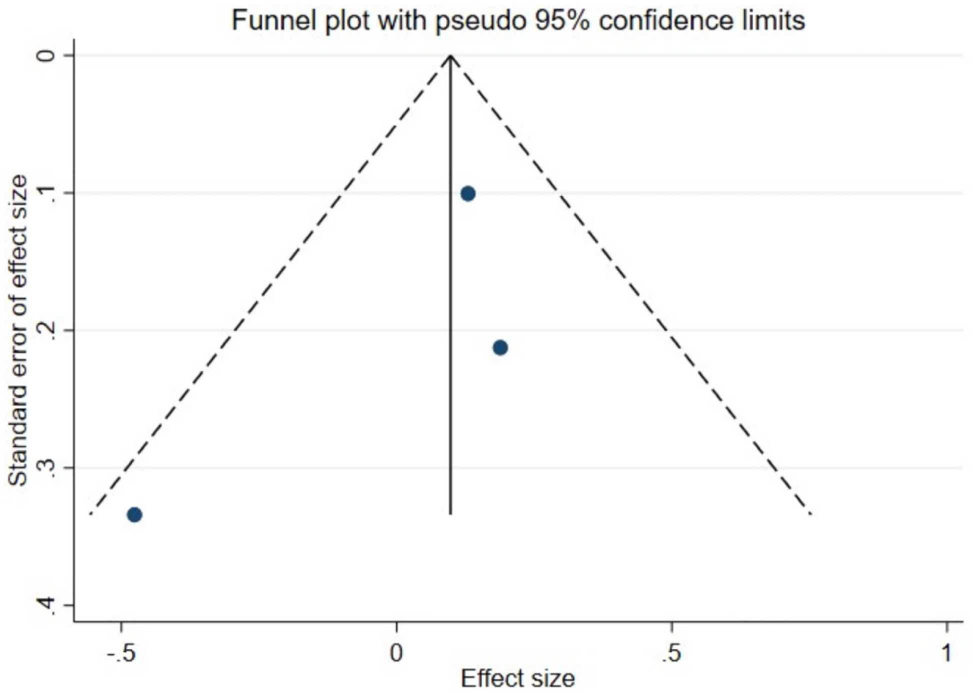

Supplementary Figure 2. Funnel plot of effect of vitamin B12 and folic acid on change in ADAS-Cog score. Egger's test (intercept = −1.61, t = −0.98, 2-tailed $p = 0.508$) and Begg's test (z = 1.04, $p = 0.296$) did not reveal any publication bias.

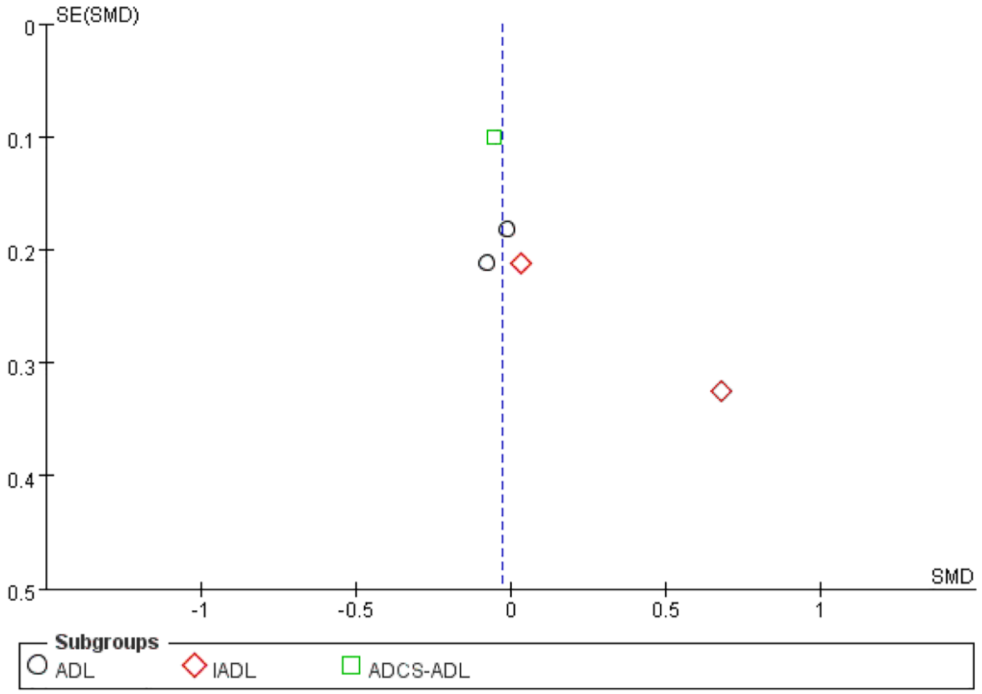

Supplementary Figure 3. Funnel plot of effect of vitamin B12 and folic acid on change in daily life functions.

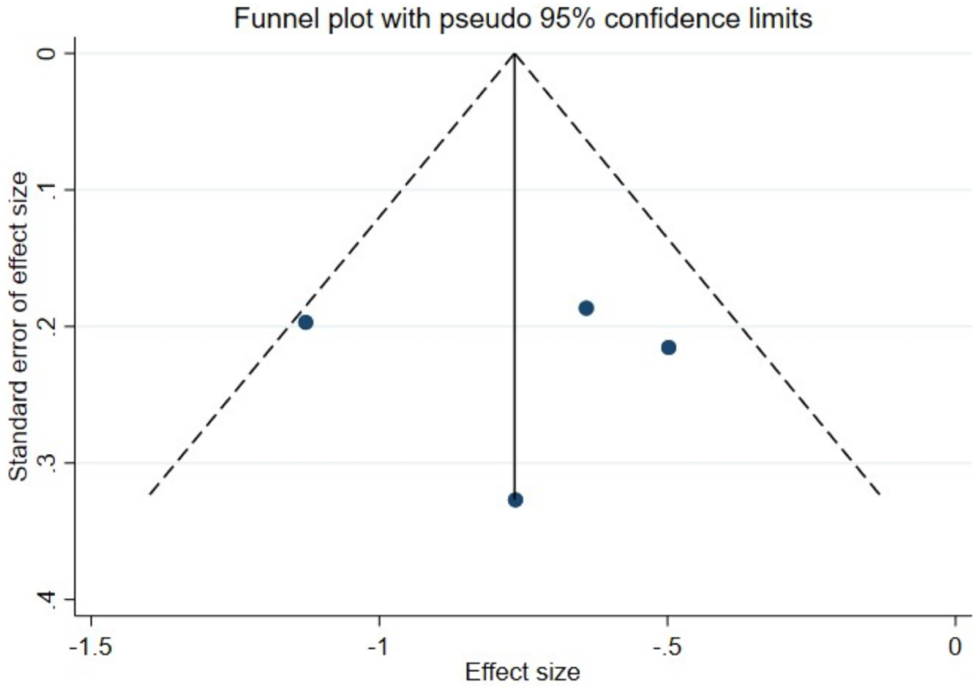

Supplementary Figure 4. Funnel plot of effect of vitamin B12 and folic acid on change in blood homocysteine level. Egger's test (intercept = 0.48, t = 0.11, 2-tailed p = 0.921) and Begg's test (z = −0.34, p = 1.00) did not reveal any publication bias.
