# Supplementary material for: Role of vitamin B12 and folic acid in treatment of Alzheimer’s disease: a meta-analysis of randomized control trials
Source: Aging (Albany NY). 2024 May 2;16(9):7856–69. doi: 10.18632/aging.205788 (PMC11132008; doi:10.18632/aging.205788)
Supplement: Supplementary Table 1 [file aging-16-205788-s002.pdf]

SUPPLEMENTARY TABLE

Supplementary Table 1. Search strategy.

**PubMed**		
1	Search: (((vitamin[Title/Abstract]) OR (vitamin B[Title/Abstract])) OR (folate[Title/Abstract])) OR (folic acid[Title/Abstract])	2600302
2	Search: ((((alzheimer[Title/Abstract]) OR (alzheimer's[Title/Abstract])) OR (dementia[Title/Abstract])) OR (cognition[Title/Abstract])) OR (cognitive[Title/Abstract])	675368
#1 AND #2	Search: #1 AND #2 ("vitamin"[Title/Abstract] OR "vitamin b"[Title/Abstract] OR "folate"[Title/Abstract] OR "folic acid"[Title/Abstract]) AND ("alzheimer"[Title/Abstract] OR "alzheimer's"[Title/Abstract] OR "dementia"[Title/Abstract] OR "cognition"[Title/Abstract] OR "cognitive"[Title/Abstract])	5646
RCT	Search: #1 AND #2 Filters: Randomized Controlled Trial (("vitamin"[Title/Abstract] OR "vitamin b"[Title/Abstract] OR "folate"[Title/Abstract] OR "folic acid"[Title/Abstract]) AND ("alzheimer"[Title/Abstract] OR "alzheimer's"[Title/Abstract] OR "dementia"[Title/Abstract] OR "cognition"[Title/Abstract] OR "cognitive"[Title/Abstract])) AND (randomizedcontrolledtrial[Filter])	361
**Embase**		
1	vitamin:ab,ti OR 'vitamin b':ab,ti OR folate:ab,ti OR 'folic acid':ab,ti	333693
2	alzheimer:ab,ti OR alzheimers:ab,ti OR dementia:ab,ti OR cognition:ab,ti OR cognitive:ab,ti	896614
#1 AND #2	#1 AND #2 (vitamin:ab,ti OR 'vitamin b':ab,ti OR folate:ab,ti OR 'folic acid':ab,ti) AND (alzheimer:ab,ti OR alzheimers:ab,ti OR dementia:ab,ti OR cognition:ab,ti OR cognitive:ab,ti)	8375
RCT	#1 AND #2 AND 'randomized controlled trial'/de	532
